# Biomimetic Synthetic Somatic Markers in the Pixelverse: A Bio-Inspired Framework for Intuitive Artificial Intelligence

**DOI:** 10.3390/biomimetics11010063

**Published:** 2026-01-12

**Authors:** Vitor Lima, Domingos Martinho

**Affiliations:** 1 ISLA Santarém-Polytechnique University, Rua Dr. Teixeira Guedes, 31, 2000-029 Santarém, Portugal; vitor.lima@islasantarem.pt; 2 NECE–Research Centre for Business Sciences, Estrada do Sineiro 56, 6200-209 Covilhã, Portugal

**Keywords:** affective computing, biomimetics, bio-inspired artificial intelligence, grid-world environments, somatic marker hypothesis, synthetic somatic markers

## Abstract

Biological decision-making under uncertainty relies on somatic markers, which are affective signals that bias choices without exhaustive computation. This study biomimetically translates the Somatic Marker Hypothesis (SMH) into synthetic somatic markers (SSMs), a minimal and interpretable evaluative mechanism that assigns a scalar valence to compressed environmental states in the high-dimensional discrete grid-world Pixelverse, without modelling subjective feelings. SSMs are implemented as a lightweight Python routine in which agents accumulate valence from experience and use a simple threshold rule (θ = −0.5) to decide whether to keep the current trajectory or reset the environment. In repeated simulations, agents perform few resets on average and spend a higher proportion of time in stable “good” configurations, indicating that non-trivial adaptive behaviour can emerge from a single evaluative dimension rather than explicit planning in this small stochastic grid-world. The main conclusion is that, in this minimalist 3 × 3 Pixelverse testbed, SMH-inspired SSMs provide an economical and transparent heuristic that can bias decision-making despite combinatorial state growth. Within this toy setting, they offer a conceptually grounded alternative and potential complement to more complex affective and optimisation model. However, their applicability to richer environments remains an open question for future research. The ethical implications of deploying such bio-inspired evaluative systems, including transparency, bias mitigation, and human oversight, are briefly outlined.

## 1. Introduction

Biomimetic approaches have proven to be very successful in translating biological principles into translation for developing efficient computational systems. Following Damásio’s somatic marker hypothesis (SMH) [1], which governs neurobiological decision-making, this study establishes synthetic somatic markers (SSMs) as a new mechanism within the artificial intelligence (AI) domain at biomimetic methodology under combinatorial uncertainty. Artificial systems that can perform adaptive, flexible, and context-sensitive behaviour remain one of the main problems in artificial intelligence (AI) and cognitive science because standard computational setups fall short in situations defined by uncertainty and combinatorial explosion. Although there have been great steps forward in deep learning and optimisation methods, these setups have structural weaknesses when dealing with environments marked by low feedback signals, high branching factors, and exponential state-space growth because exhaustive assessment becomes computationally possible. In these scenarios, exhaustive analysis becomes impractical, and optimisation methods face structural limitations arising from the problem of dimensionality, where the space of states grows exponentially while the scarcity of data reduces the effectiveness of learning [2].

However, human cognition relies on additional regulatory mechanisms that facilitate decision-making even under deep uncertainty, suggesting that adaptive intelligence is not based exclusively on logical deliberation. Damasio et al. [1,3] demonstrated that individuals with prefrontal lesions face significant difficulties in daily decision-making despite preserving their logical-analytical capacity. This dissociation motivated the formulation of the SMH, which postulates that physiological states encode evaluative signals, called somatic markers, which restrict the decision space by attributing affective value to potential options before conscious reasoning occurs [4].

Recent studies have expanded this perspective by demonstrating that evaluative and interoceptive signals are computationally efficient heuristics that integrate perception, memory, and expectation, thereby reducing the complexity of decision-making under uncertainty [5,6]. Similarly, constructivist approaches to emotion conceive of affective states as predictive inferences based on expectation, context, and internal modelling [7]. Affective heuristics provide a complementary framework in decision psychology: rapid emotional evaluations function as decision shortcuts that are adaptive to uncertainty but avoid complex calculations [8].

At the same time, affective computing has shown that emotion-inspired mechanisms can improve the adaptability and robustness of artificial systems, even without involving any form of subjective experience [9].

More recent reviews describe a shift towards multimodal and hybrid architectures that integrate visual, auditor, and physiological signals and rely on increasingly complex fusion strategies for affect recognition and regulation [10,11]. In parallel, surveys of emotion in reinforcement learning (RL) agents and robots systematise how emotion-like variables, derived from reward, value, or homeostatic processes, can modulate learning efficiency and action selection [12].

In recent years, research on the computational mechanisms of emotion and evaluative processes has intensified, as shown by recent surveys that systematise emotion models across symbolic, statistics, and embodied AI approaches [6,13]. Reviews of affective computing highlight an evolution towards hybrid models that combine multimodal perception, internal simulation, and context-sensitive heuristics [10,14]. The integration of AI into real-world environments has demonstrated that evaluative systems can improve behavioural adaptation under uncertainty, although challenges remain regarding emotional variability, robustness, and explainability [15]. Ethical concerns related to privacy and the use of physiological data, especially in sensory devices and affective recognition, have been highlighted in additional studies [10].

Bio-inspired approaches have introduced artificial somatic markers and somatic indices as compact, experience-based variables that regulate decision-making in autonomous systems, suggesting that somatic-like evaluations can be operationalised in artificial agents [16].

More recently, Somatic Q-Learning explicitly integrates a somatic-marker-inspired signal into a RL framework, further supporting the viability of SMH-based evaluative mechanisms in artificial agents [17].

These developments collectively motivate the exploration of biologically inspired evaluative mechanisms, such as synthetic somatic markers (SSMs), which can operate as lightweight, interpretable, and computationally economical heuristics for regulating behaviour in uncertain and dynamically unstable environments, in line with broader proposals that treat self-concern and somatic-like evaluations as core motivational principles in artificial agents [18].

In this context, investigating minimalist evaluation mechanisms that can serve as computationally economical alternatives to conventional models is particularly relevant. This line of research suggests that integrating bio-inspired heuristics is a promising avenue for enhancing the adaptability and interpretability of artificial systems.

Accordingly, the present study has two complementary objectives: (i) to articulate a theoretical and methodological foundation demonstrating how SMH-inspired evaluative mechanisms can support interpretable AI, and (ii) to empirically show, through a minimal computational model, how such mechanisms can generate functional behaviour analogous to intuition.

Existing affective computing and emotion in RL models tend to rely on complex, often multimodal architectures, high-dimensional internal state representations, and task-specific design choices, which can hinder transparency, reproducibility, and deployment in resource-constrained settings. Bio-inspired approaches that implement artificial somatic markers demonstrate the usefulness of evaluative shortcuts but typically embed them in richer agent architectures or domain-specific control systems, making it harder to isolate the core contribution of somatic-like evaluation itself. Consequently, the extent to which a single, low-dimensional SMH-inspired evaluative signal can go in supporting adaptive behaviour under combinatorial explosion when decoupled from complex perception and learning pipelines. This study explores how a single scalar evaluative signal under combinatorial explosion within a minimal 3 × 3 Pixelverse, rather than providing a general solution for decision-making under uncertainty.

Accordingly, this study makes the following contributions:

- Conceptual: SSMs are introduced as a biomimetic operationalisation of the somatic marker hypothesis via a single scalar valence over compressed grid-world states (Pixelverse).
- Methodological: This study provides a minimal, fully transparent Python 3.13 implementation coupling Pixelverse, the SSM mechanism, and a threshold-based agent for reproducible evaluation under combinatorial explosion.
- Empirical: This single evaluative dimension reduces environment resets and increases time in stable “good” configurations across repeated simulations, yielding a rudimentary experience-based bias in keep/reset decisions in this minimal setting rather than full-fledged synthetic intuition.
- Integrative/ethical: Positions SSMs relative to affective computing and emotion in RL models and outlines key ethical implications, including transparency, bias mitigation, and human oversight.

The remainder of this article is organised as follows. Section 2 presents the conceptual framework and the Pixelverse environment; Section 3 details the computational model; Section 4 discusses the results; Section 5 addresses ethical considerations; Section 6 concludes the study; and Section 7 outlines directions for future research.

## 2. Conceptual Framework

This conceptual framework aims to systematise the main concepts, theories and models that underpin this study. Through a rigorous hierarchical organisation, it aims to integrate elements of neuroscience, cognitive psychology and computer science that support the SSMs proposal.

The core components of the model are detailed below, starting with a description of the Pixelverse as a computational environment, followed by an explanation of the SMH, and finally the formulation of the SSMs.

### 2.1. Pixelverse a Computational Environment

The Pixelverse is a high-dimensional mathematical field composed of discrete pixels of microstates, each of which can assume a finite number of values. Although visually simple, each pixel functions as a local computational entity governed by transition rules, neighbourhood interactions, and stochastic fluctuations. Thus, even minimal configurations produce astronomically large state spaces. A grid of only 3 × 3 pixels, each capable of taking on two colours, generates 2^9^ = 512 possible states. Extending the grid to 6 × 6 pixels already yields $2^{36}=68,719,476,736$ possible states, illustrating how even modest increases in grid size lead to intractable state spaces. This situation illustrates the canonical explosion problem in state space, which is common in biomimetic design processes [19]. Even minimal local structures can generate computationally intractable global dynamics, reinforcing the need for heuristic and evaluative mechanisms rather than exhaustive search.

The Pixelverse used in this work adopts a conceptual inspiration similar to the spaces explored in generative computational art, where simple local rules produce highly complex global dynamics. Lima’s [20] original observation that even small pixel grids produce combinatorial intractability serves here as an intuitive entry point: the Pixelverse is not a visual object but a formal environment whose complexity increases exponentially and unpredictably.

In this environment, classic AI techniques, which rely on explicit optimisation, exhaustive evaluation, or deep tree research, become ineffective. Even RL systems face difficulties as scarce rewards, stochastic transitions, and high branching factors make convergence difficult. Therefore, a mechanism that allows an intelligent agent to act on incomplete information, guided not by a perfect prediction but by a rapid heuristic evaluation, is necessary. This sets the stage for the introduction of synthetic evaluative mechanisms inspired by human neurocognitive architecture.

### 2.2. Somatic Marker Hypothesis as a Guiding Principle

The SMH provides a powerful neurobiological model for such an evaluation mechanism. According to the SMH, emotional and physiological responses encode valence signals, which are somatic markers that influence decision-making in situations of uncertainty or high complexity [1]. These markers do not replace cognition; however, they restrict the decision space, allowing faster and more adaptable behaviour.

The classic empirical basis for SMH comes from the Iowa Gambling Task (IGT), in which healthy individuals develop anticipatory physiological responses to disadvantageous choices before they can consciously articulate the underlying rule structure [4]. In contrast, patients with damaged ventromedial prefrontal cortex do not develop these anticipatory markers and consistently make wrong decisions despite having intact logical faculties [1]. This finding showed that somatic markers act as prelogical evaluative shortcuts, helping the brain avoid harmful trajectories without calculating all possible future states.

Contemporary research continues to consolidate and refine SMH. Lin et al. [21], highlight how emotional-physiological evaluations remain central to decision-making under conditions of uncertainty. Duplessis-Marcotte et al. [22] revisited the SMH using multilevel models and confirmed that somatic markers continue to shape behavioural trajectories in dynamic choice contexts. These studies confirm the central claim of SMH: intelligent behaviour arises from the interaction between emotion and cognition, not just logic. This makes SMH particularly suitable for minimalist computational environments such as the Pixelverse, where agents must rely on compressed evaluative signals rather than full predictive modelling. The SMH formalises this principle: affective markers operate as low-dimensional heuristics that guide behaviour when the underlying state space is too large to reanalysed exhaustively.

Thus, SMH provides a functionally and computationally relevant principle: emotional assessments act as compressed representations of past experiences, reducing complexity by providing rapid valence assessments. This mechanism is used by artificial agents when navigating environments such as the Pixelverse.

Thus, somatic markers function as evaluative compression mechanisms rapid and economical representations of accumulated experiential value, a principle directly operationalised by the computational model presented in this article.

### 2.3. Synthetic Somatic Markers

SSMs is an evaluative mechanism that combines state compression, cumulative valence, and threshold decision, integrating representation, evaluation, and action into a single functional scheme. This operationalisation differs from traditional artificial emotional approaches in that it focuses exclusively on the adaptive evaluative function without resorting to affective simulation, emotional recognition, or multimodal mappings.

Inspired by SMH, we propose SSMs. SSMs are computational subroutines that assign evaluative scalar values to specific states of the Pixelverse without implying any real emotional phenomenon. This scalar value is only a functional proxy for ‘feeling’ in the narrow sense of compressed evaluative information and should not be interpreted as a model of subjective experience.

An SSMs can be defined as a valence function:

v:S→[−1,1]

where S represents the set of all possible states of the Pixelverse, and v(s) functions as an evaluative experience accumulator updated through a simple but expressive incremental rule. This mechanism transforms valence into a condensed record of the agent’s interactive history, reflecting the system’s tendency to reinforce and penalise stable states associated with unfavourable outcomes.

The value v(s) is adjusted iteratively based on previous outcomes: states that lead to stability, predictability, or rewards accumulate positive valence, whereas states that precede instability, excessive noise, or system “collapse” accumulate negative valence. This process generates an emergent mapping between experience and evaluation, which is loosely analogous to how repeated outcomes shape evaluative biases in biological cognition.

When an agent explores the Pixelverse by encountering an *s* state, the synthetic marker queries the accumulated history and returns a valence value that summarises the experience. A high positive valence acts as a compact indicator that the corresponding region of the state space has been historically favourable, encouraging the agent to stay in or seek out similar states. Conversely, a negative valence plays the role of an “alarm signal”, leading the agent to restart the environment, avoid similar trajectories, or adopt corrective behaviours.

This mechanism allows quick action without the agent having to simulate all possible trajectories. These shortcuts are not arbitrary: they are based on experience, shaped by environmental feedback, and updated with each iteration.

Cabrera-Paniagua et al. [16] explored the feasibility of using artificial somatic markers as standalone agents. They proposed a framework to incorporate artificial somatic markers into all decision-making phases, demonstrating that evaluative shortcuts increase adaptability in uncertain environments. Although structurally different from the approach proposed in this work, their results confirm that bio-inspired evaluation mechanisms can improve decision-making.

Thus, SSMs constitute a computational analogue of the SMH, allowing artificial agents to navigate the combinatorial complexity of the Pixelverse using experience-based adaptive evaluative heuristics. Recent models of artificial emotional intelligence demonstrate that distinct evaluative representations can offer broad expressiveness with low computational complexity [22].

### 2.4. Relation to Affective Computing Models

Since Picard’s formulation [9], affective computing has focused mainly on providing systems with the ability to recognise, model, and sometimes express human emotions, typically using multimodal inputs (facial expression, voice, physiology) and explicit representations in terms of emotional categories or dimensions such as valence activation. In contrast, the present SSM model does not target any form of affective recognition or emotional interaction with users: it implements a single intrinsic valence signal to the task, defined on the Pixelverse’s compressed signatures, which functions only as an evaluative heuristic for decisions to maintain or restart in combinatorial explosion scenarios. 

In a complementary way, the emotion models in RL reviewed by Moerland et al. [12] treat emotional states (e.g., fear, hope, or surprise) as variables derived from reward, value, or homeostasis, which directly modulate the learning dynamics and social behaviour of agents and robots. 

In this work, SSMs are conceptualised as a minimal biomimetic evaluative layer, inspired by the somatic marker hypothesis, which can be coupled to RL algorithms in principle. However, this study examines it in isolation to illustrate how a single synthetic evaluative dimension can bias exploration in a small, stochastic 3×3 grid-world, rather than to claim a general solution for decision-making under extreme uncertainty.

In this sense, SSMs are designed as an evaluative component that can, in principle, be combined with standard RL algorithms rather than replacing them, so that future benchmarks can test whether they improve adaptability or interpretability when used alongside established methods.

Table 1 presents the synthesis of the main differences between affective models.

## 3. Computational Model

The developed computational model operationalises the concepts presented in the conceptual framework and makes explicit the relationship between SMH-inspired neurocognitive principles and a minimal computational pipeline. The objective of this study is to demonstrate, in a transparent and reproducible manner, how an artificial agent can develop a functional evaluative mechanism that guides decisions in a small stochastic grid-world exhibiting combinatorial state growth without claiming a general solution for decision-making under uncertainty.

**Figure 1:**
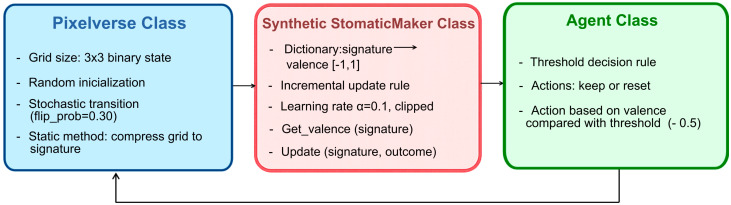
Schematic overview of the proposed methodology: interaction loop between Pixelverse, SSMs, and the threshold-based agent.

Concretely, the model couples three minimal components in a closed interaction loop: (i) the Pixelverse, a stochastic grid-world environment; (ii) a synthetic somatic marker (SSM) that stores a scalar valence for each compressed state signature; and (iii) a threshold-based agent that selects actions based on this valence. At each time step, the agent observes the current grid, compresses it into a signature, retrieves its valence from the SSM, chooses either to keep the state or reset the environment according to a fixed threshold, receives an outcome label (good vs. bad) from a simple stability rule, and updates the corresponding valence value clipped to the interval [−1,1] (Figure 1). This interaction cycle implements the proposed SMH-inspired evaluative heuristic and is detailed in the following subsections.

### 3.1. General Structure of the Pixelverse

**Figure 2:**
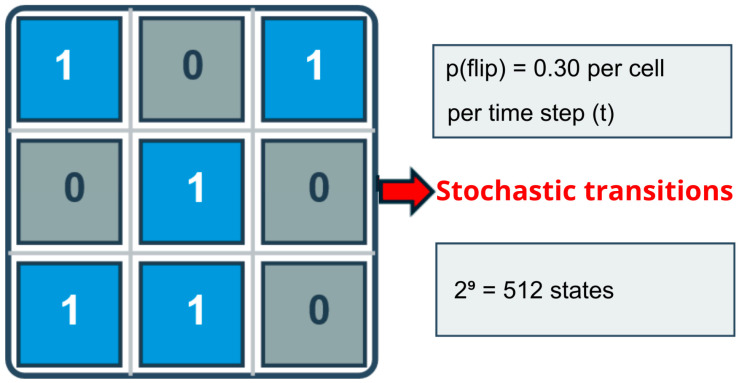
Pixelverse Structure 3 × 3.

As illustrated in Figure 2, the Pixelverse is formalised as a two-dimensional grid of dimension n × n, where each cell assumes a binary state {0,1}.

In the presented simulations, n = 3 was used, which generates a state space with possible configurations that are 2^9^ = 512 sufficient to explicitly illustrate the combinatorial explosion in an extremely minimalist scenario. The environment’s temporal evolution occurs through stochastic transitions: each cell reverses its state with a fixed probability (as implemented: 0.30) at each time step, which is common to the entire grid. Thus, the global state at time t, is $P_{t}$ a binary matrix subject to fluctuations that introduce uncertainty and make explicitly predicting trajectories virtually impossible.

The state space of the Pixelverse grows exponentially with $2^{n^{2}}$, making decision approaches based on enumeration or advanced planning unfeasible as n increases. The n = 3 case is used here as a prototypical scenario to test minimalist evaluative mechanisms: the agent must decide without knowing all future consequences, in line with the idea that decisions under uncertainty resort to evaluative shortcuts rather than exhaustive calculation [3]. The choice of this simple environment is deliberate, allowing us to isolate and clearly observe the specific contribution of synthetic valence mechanisms in a fully transparent, small-scale setting.

### 3.2. Compression and Internal Representation of State

Given the space intractability, $2^{n^{2}}$ the agent does not operate directly on the complete Pixelverse matrix. Instead, it uses a compressed representation of the state, called a signature $\sigma (P_{t})$, which functions as a partial and reduced mapping of the environment, preserving only the characteristics considered relevant for heuristic evaluation.

The compression used in this prototype is based on counts per line following the following rule:

$$\sigma (P_{t})=(r_{1},r_{2},\ldots ,r_{n}),$$

where $r_{i}\;$ represents the number of active cells in a row i. Although minimalist, this form of compression preserves the overall density information and some structure, allowing it to distinguish between sparse, dense, or balanced states. Compression is intrinsically non-invertible: different Pixelverse arrays can share the same signature σ. This controlled loss of information brings the agent closer to a realistic cognitive condition in which the perception is partial and redundant details are discarded. This approach is consistent with computational rationality and evaluative computation approaches that treat affective evaluations as compressed representations of experience [6,22].

### 3.3. Valence Update Mechanism

The functional core of SSMs lies in assigning a scalar valence value to each state signature. The valence function V(σ) is initialised to 0 for any not yet observed signatures and is updated exclusively when the corresponding signature is visited according to the following rule:

$$V_{t+1}\left(\sigma_{t}\right)=\mathrm{clip}\left(V_{t}\left(\sigma_{t}\right)+\alpha \cdot {\mathrm{result}\;}_{t},\;-1,1\right),$$

where α=0.1 denotes the evaluative learning rate and result∈{−1,+1} indicates whether the new state was classified as “bad” (−1) or “good” (+1). The clipping function ensures that $v_{s}$ remains within the interval [−1,1]. This incremental update follows the spirit of value-updating rules in RL, where a scalar evaluation is adjusted by a learning-rate-scaled outcome term [23], and implements an oracle-guided accumulation of scalar valence over compressed signatures, given externally provided good/bad labels and is consistent with emotion in RL models in which emotion variables track recent rewards and prediction [12]. The update occurs locally for each signature s, independently of all other signatures. Thus, the valence becomes a cumulative record of the synthetic affective experience agent’s, functioning as a computational analogue of somatic markers as evaluative summaries of past experience described in SMH [1].

### 3.4. Agent Decision Architecture

At each time step, the agent performs two actions: maintain the current environment state or restart the Pixelverse. The decision is based on the valence associated with the current state signature through $\sigma_{t}$ the following threshold rule:

$$a_{t}=\{\begin{array}{cc} \mathrm{reset}, & \mathrm{if}\;V(\sigma_{t})<\theta ,\\ \mathrm{keep}, & \mathrm{otherwise}, \end{array}$$

θ = −0.5 represents the structural risk aversion level. More negative values of θ would make the agent more tolerant of historically unfavourable states, whereas higher values would increase sensitivity to the evaluative history.

In all reported simulations, the decision threshold was set to θ=−0.5.

This simple threshold rule corresponds to a sign-based decision heuristic, analogous to fixed-bound mechanisms in drift-diffusion and signal detection models, where an internal variable triggers choices once a decision boundary is crossed by a decision boundary [24].

### 3.5. Interaction Cycle and Behaviour Emergence

The agent interacts with the environment through a fixed sequence of operations at each time step t. First, the Pixelverse grid is observed and transformed into a compressed signature *σ*(*P_t_*). This signature is used to retrieve the corresponding valence *V*(*σ_t_*), reflecting the agent’s cumulative evaluative experience. Based on this value and the decision threshold θ, the agent selects one of two possible actions: keep or reset.

The stochastic transition function updates the environment when the agent selects keep, generating a new grid P_t+1_. This new configuration is evaluated using the predefined stability rule: states with three or fewer active cells are classified as good (+1), whereas all others are classified as bad (−1). This stability rule acts as an externally specified oracle that assigns “good” or “bad” labels to each visited grid configuration; the agent does not infer or adapt this evaluative criterion. When the agent selects reset, the environment is reinitialized, and the outcome is set to −1, representing the cost of abandoning the current trajectory.

However, we note that, even under this externally specified criterion, the agent does not receive any explicit policy or model of the environment and must still construct an internal evaluative map over compressed signatures. In this sense, the present study isolates oracle-guided accumulation of scalar valence over compressed states, rather than strong evaluative learning in which the value structure itself is discovered.

The resulting outcome is then used to update the valence associated with *σ*(*P*_*t*_) according to the incremental rule described in Section 3.3. All variables involved in the iteration (t, σ_t_, V(σ_t_), action, and outcome) are recorded in the simulation log for subsequent analysis. Over time, this cyclic process leads to the emergence of adaptive behaviour: the agent increases the frequency of keep decisions in historically favourable regions of the state space while reducing resets in unstable configurations.

This dynamic illustrates how a minimal evaluative mechanism can generate adaptive behaviour in this small stochastic grid-world without requiring explicit prediction or exhaustive modelling, suggesting a possible heuristic role for such mechanisms under uncertainty while remaining restricted to the present toy setting [3,6].

The general procedure is summarised in pseudocode (Algorithm 1).
**Algorithm 1.** Pixelverse–SSM interaction loop
1. Initialise Pixelverse environment; generate initial grid.
2. Initialise SSM memory with valence $v_{s}=0$ for all signatures s.
3. For each time step t=1…T:
   a. Observe current grid and compute compressed signature s=compress(grid).
   b. Retrieve valence v=SSM.get_valence(s).
   c. Agent selects action a∈{keep,reset} using threshold rule:
    If v<θ then a=reset.
    Else a=keep.
   d. If a=reset:
    reinitialise grid; set outcome = −1.
    Else (a=keep):
    update grid using stochastic transition;
    count active cells; set outcome = +1 if active ≤ 3, otherwise outcome = −1.
   e. Update valence for signature s:
    v←v+α⋅outcome;
    clip v to [−1,1]; store updated v in SSM memory.
   f. Log (t,s,v,a,outcome) for later analysis

### 3.6. Computational Simulation Protocol

The proof-of-concept is implemented as a numerical simulation that couples the Pixelverse environment with the SSM mechanism and the decision-making agent. Each run consists of a sequence of iterations on a 3 × 3 binary grid, which is sufficient to make the combinatorial explosion explicit while keeping the dynamics fully inspectable. The implementation relies only on widely available standard libraries (in particular random and data classes), reinforcing the goal of transparency and ease of replication

The code is organised into three main classes (Pixelverse, SyntheticSomaticMarker and Agent), following biomimetic design modularity [20]. The Pixelverse class generates an initial random 3 × 3 grid and updates it using a stochastic transition rule in which each cell flips its state with a fixed probability (flip_prob = 0.30) at every time step. A static method compresses the entire grid into a reduced signature, counting the number of active cells in each row, resulting in a compact representation that preserves the overall density information while remaining many-to-one. The SyntheticSomaticMarker class maintains a dictionary that maps each observed signature to a scalar valence in the range [−1, 1], updated through an incremental rule with learning rate α = 0.1 and clipped to this range. The Agent class implements a threshold-based decision rule with two available actions at each step: keeping the current environment or resetting the Pixelverse to a new random grid. For a given signature, the agent queries its associated valence and compares it with a negative threshold (e.g., −0.5); if the valence falls below this threshold, the agent chooses to reset; otherwise, it maintains the current state. In this proof-of-concept setting, θ, α, and flip_prob were initially chosen based on qualitative exploration to obtain rich yet numerically stable dynamics. Subsequently, we conducted a small parameter sensitivity analysis around these defaults, varying α, θ, and flip_prob to assess how robust the qualitative behaviour of the SSM agent is to such changes.

This simple architecture emphasises that non-trivial regulation of behaviour can emerge from a single evaluative dimension combined with a fixed risk-aversion parameter.

At each iteration, the simulation executes the following cycle: (i) compute the compressed signature of the current grid, (ii) retrieve the corresponding valence, (iii) select the action to keep or reset according to the threshold rule, (iv) update the environment either by stochastic transition (keep) or by random reinitialization (reset), (v) classify the resulting grid as good or bad, and (vi) update the valence associated with the previous signature. 

For each run, the simulation records a record containing the time index, the compressed signature, the valence value before the update, the chosen action, and the resulting result in each time step. The aggregate metrics of this logarithm include the total number of environmental resets, the temporal evolution of the valence, the relative frequency of good states, and the empirical distribution of the signatures visited.

In addition to the SSM-based agent, three baseline agents were implemented in the same Pixelverse environment, under the same externally defined stability rule. First, a random policy agent selects keep or reset at each time step with fixed probabilities calibrated to match the original SSM agent’s marginal reset rate. Second, a frequency-based agent without valence accumulation stores the empirical frequency of “good” outcomes for each compressed signature and chooses to keep this frequency exceeds a fixed threshold (e.g., 0.5), resetting otherwise. Third, a minimal tabular Q-learning agent maintains Q-values over signatures and the two actions (keep, reset), updating them with a standard Q-learning rule using fixed learning-rate and discount parameters. All agents were evaluated under the same simulation protocol as the SSM agent (30 runs, 100 iterations per run, distinct random seeds), and the same aggregate metrics were reported across runs: total number of resets, proportion of “good” states, and statistics for the most frequently visited signatures.

To assess robustness, we ran the simulation 30 times with 100 iterations each and different random seeds, and computed mean ± standard deviation for the total number of resets, the proportion of ‘good’ states (active cells ≤ 3), and the average valence of the three most frequently visited signatures.

This minimalist implementation exemplifies the principles of biomimetic design: simple local rules generating complex adaptive global behaviours, mirroring biological efficiency [25]. The Python source code implementing the Pixelverse dynamics, the SSM mechanism, and the baseline agents used for the comparisons in Section 4.2 is provided as Supplementary Material S1 and S2.

## 4. Results and Discussion

### 4.1. Main SSM Behaviour in the 3 × 3 Pixelverse

The numerical results obtained in the Pixelverse simulations show that SSMs operate as simplified evaluative mechanisms that enable agents to develop adaptive behaviours in this small stochastic grid-world, which exhibits combinatorial state growth, without claiming a general solution for decision-making under uncertainty.

Across 30 independent runs with 100 iterations each and different random seeds, the agent performed on average 1.7±2.4 resets and spent 0.26±0.07 of time steps in ‘good’ states (active cells ≤ 3), while the average valence of the three most frequently visited signatures stabilised around −0.38±0.22 (Table 2). These repeated-run statistics indicate that the qualitative pattern of reduced resets and differentiation between more and less favourable regions of the state space is robust rather than a single-run artefact.

A consistent reduction in the frequency of environment resets is observed, while the permanence of stable states, reflecting the specialisation of the valence function in distinguishing between favourable and unfavourable configurations [3,4].

### 4.2. Baseline Comparisons

Beyond the SSM agent alone, we also compared its behaviour to the three baseline agents introduced in Section 3.6. The random policy agent exhibited substantially higher reset counts and a lower proportion of “good” states than the SSM agent across the same protocol of 30 runs with 100 iterations each, reflecting the absence of any evaluative memory. The frequency-based agent without valence accumulation partially reproduced the tendency to favour historically better signatures, but its performance remained below that of the SSM agent on both resets and time in “good” states, and the average valence of the most frequently visited signatures was less stable. The minimal Q-learning agent approached SSM-like performance only under carefully tuned learning and discount parameters, while relying on a less transparent internal evaluation structure. These comparisons (Table 3) suggest that the SSM mechanism is not reducible to a trivial artefact of memory and thresholding, although its empirical support remains restricted to the present toy environment.

Table 3 shows that the SSM agent performs far fewer resets than the random policy and the Q-learning agent, while spending a larger proportion of time in “good” states than all baselines, despite relying only on a single scalar evaluative signal. In contrast, the frequency-based agent without valence accumulation degenerates into constant resetting in this setting (100 ± 0.00 resets per run), never stabilising on favourable configurations. Taken together, these results support the claim that the SSM mechanism yields non-trivial adaptive behaviour relative to simple memory-free or purely frequency-based heuristics, while offering a more transparent evaluative structure than tabular Q-learning in the same toy environment.

### 4.3. Parameter Sensitivity

To explore the robustness of the SSM mechanism, a small sensitivity analysis was conducted around the default parameter settings. Specifically, we varied the learning rate, α ∈ {0.05,0.10,0.20} the decision threshold, and the flip probability flip_prob ∈ {0.20,0.30,0.40} while keeping the other parameters fixed at their default values. For each configuration, we ran 30 simulations of 100 iterations and computed the same summary metrics as in Table 2: total resets per run and proportion of “good” states (active cells ≤ 3). Across moderate variations (e.g., α = 0.05 or 0.20, θ = −0.3 or −0.7, flip_prob = 0.20 or 0.30), the qualitative pattern observed in the default setup was preserved: the SSM agent still performed relatively few resets and spent a non-trivial fraction of time in “good” configurations, while maintaining a clear differentiation between more and less favourable regions of the state space. In contrast, more extreme settings (for exemple, flip_prob = 0.40) combined with a relatively lax threshold) led to more frequent resets and less stable valence profiles, indicating that the current mechanism operates best within a bounded parameter regime rather than being uniformly robust.

### 4.4. Failure Modes and Maladaptive Regimes

Documenting situations in which the mechanism performs poorly is important. In additional exploratory runs, the same evaluative machinery can produce qualitatively different maladaptive patterns.

In some parameter settings, the agent tends to reset the environment too frequently, particularly when flip probabilities are relatively high and decision thresholds are more permissive, abandoning trajectories before any stable evaluative structure can emerge. Under these conditions, the number of resets increases markedly, and the proportion of “good” states remains low, suggesting that the evaluative layer fails to establish robust preferences over compressed signatures.

Other α ∈ {0.05,0.10,0.20} regimes reveal a complementary problem, in which the interaction between the environment dynamics and the valence update rule favours recurrent visits to a small subset of unfavourable configurations. Here, the agent can effectively become trapped in low-valence cycles, repeatedly revisiting signatures that accumulate negative valence without escaping towards alternative regions of the state space, so that the same mechanism that stabilises successful patterns can also lock in maladaptive ones.

A further difficulty arises from the compression scheme itself: because multiple distinct grids are mapped onto the same signature, some configurations are evaluatively different under the oracle rule but indistinguishable for the agent. In such cases, signatures aggregating both good and bad grids lead to noisy valence estimates and inconsistent keep/reset decisions. These failure modes highlight structural limitations of this design—coarse compression, one-dimensional valence, and a fixed threshold—and directly motivate the extensions discussed in Section 7.

### 4.5. Conceptual Implications

This behaviour evokes the evaluative heuristics described in the neuroscientific literature, in which rapid evaluations based on affective signals function as effective decisive shortcuts, restricting the space for action and favouring adaptive efficiency [5,8]. Synthetic valence acts as a compact marker that summarises the accumulated experience of the agent, allowing decisions that favour predictable trajectories and penalise dysfunctional patterns without requiring exhaustive modelling or advance calculation [6].

Conceptually, we use the vocabulary of somatic markers and intuition only in this restricted functional sense, to emphasise the role of compressed evaluative signals, and we do not claim to model rich cognitive-affective states or subjective feelings.

The results reinforce the relevance of the integration between neuroscience and AI, showing that principles inspired by somatic markers can enrich the adaptability and interpretability of intelligent systems. This approach offers a conceptual alternative that may complement classical learning paradigms. However, its current empirical support is restricted to the minimal Pixelverse, and whether similar benefits arise in more complex environments remains to be tested [21].

### 4.6. Limitations

It is essential to recognise the limitations of the model, which include its conceptual simplicity, the restriction of valence to a single dimension, and the use of a minimalist environment, factors that affect the expressiveness and immediate applicability of the results [10]. All experiments are restricted to a 3 × 3 Pixelverse; the behaviour of SSMs in larger grids or more realistic environments is unknown and must be studied in follow-up work. In addition, the present agent does not learn or revise its notion of success: the good/bad classification of states is hand-crafted and acts as an external oracle. Thus, the model is best understood as an oracle-based evaluative layer rather than as strong evaluative learning in which the value structure itself is discovered.

The Pixelverse was designed on a small scale to allow for clear isolation of the contribution of the evaluative engine. This minimalism delimits the prototype’s limitations and possible applications: the objective is not to model human affection but rather to highlight the functionality of evaluative heuristics in the context of combinatorial explosion. Therefore, the present work should be understood as a minimal proof of concept rather than a demonstration of performance in complex real-world environments, clarifying that the suitability of SSMs for richer uncertain environments remains an empirical question for future studies.

Despite these constraints, they do not compromise the validity of the proof of concept and, on the contrary, indicate promising paths for future extensions, such as the incorporation of multidimensional evaluative competencies or integration with traditional RL techniques [6,8].

In summary, this work should be read as a minimal proof-of-concept rather than a general solution for decision-making under uncertainty. Within a fully transparent 3 × 3 Pixelverse, SMH-inspired SSMs operate as a simple scalar evaluative layer that can bias keep/reset decisions in the presence of stochastic dynamics and combinatorial state growth. Any extension of these patterns to larger, task-rich environments must be addressed in future work and cannot be claimed on the basis of the current micro-scale testbed alone.

## 5. Ethical Considerations

The introduction of neuroscience-inspired assessment mechanisms, such as SSMs, raises important ethical questions that require careful consideration. In particular, biomimetic systems present unique challenges: synthetic “feelings”, although grounded in biological plausibility, can lead to anthropomorphic misinterpretations by users during human-AI interaction. It is crucial to explicitly communicate that SSMs do not experience genuine emotions but rather function as evaluative computational processes. This distinction helps prevent misinterpretations, a risk that is well documented in the literature on human–machine interaction and AI systems [21,26]. 

Although SSMs are inspired by biological somatic markers and the SMH [1,4], they operate without subjective experience. Clarifying this non-emotional nature promotes transparency and supports responsible design in biomimetic AI systems, as emphasised in studies on anthropomorphism and ethical AI [26,27].

Transparency and auditability are fundamental ethical requirements. Detailed records of valence values and their updates should be kept to allow for traceability, accountability, and possible decision challenge. This is critical in domains with safety concerns or social impact, aligning with broader calls for AI explainability and audit mechanisms [28,29].

SSMs learn incrementally from experience, and systematic biases in data or environmental feedback can lead to the formation of skewed valence patterns. Such risks parallel recognised fairness challenges in machine learning, underscoring the need for continuous monitoring, validation, and correction processes to ensure equitable and reliable performance [30].

As agents’ autonomy and complexity grow, preserving human oversight mechanisms becomes indispensable, particularly in high-stakes or sensitive contexts. Human-in-the-loop or human-on-the-loop frameworks help maintain safety, accountability, and ethical governance [31].

SSM-based systems for socially sensitive applications, such as surveillance, healthcare, education or affective computing, require robust regulatory frameworks and ethical safeguards. Vulnerable or impressionable users must be protected against potential manipulation, privacy violations, and amplified social inequalities [15].

Finally, the minimalist and prototypical nature of the presented model not only facilitates a focused study of evaluative mechanisms but also demands strong ethical frameworks when expanding towards real-world, complex applications. To ensure that synthetic somatic markers contribute to trustworthy AI systems and beneficial social outcomes, the key principles for responsible deployment include transparency, continuous validation, human oversight, fairness, and accountability.

## 6. Conclusions

This study investigated, in a minimal 3 × 3 Pixelverse testbed, whether a computational SMH analogue could bias decision-making in artificial agents facing stochastic dynamics and combinatorial state growth. To the best of our knowledge, this is one of the first experimental implementations of an SMH-inspired evaluative mechanism in a grid-world environment explicitly constructed to highlight combinatorial explosion, although its empirical support remains restricted to this toy setting [1].

By introducing the Pixelverse as a formal environment of exponential state growth and proposing SSMs as lightweight evaluative mechanisms, we present a minimal model that demonstrates how cumulative evaluative heuristics can guide decisions without resorting to exhaustive modelling [8].

Empirical analysis reinforces this conceptual formulation. Across 30 independent runs, the SSM-based agent performed an average of 1.7 resets per run and spent approximately 26% of time steps in “good” states, with a stable negative valence profile for the most frequently visited signatures, indicating a robust differentiation between favourable and unfavourable regions of the state space (Table 2).

The emergence of this rudimentary synthetic intuition in our minimal setting suggests that biologically inspired evaluative heuristics may contribute to a basic layer of adaptive intelligence in simple artificial systems, although stronger claims about natural or more complex artificial agents go beyond the scope of the present study.

This finding parallels the functional role of somatic markers in biological cognition, where emotionally charged assessments provide quick shortcuts to navigate uncertainty [1,4]. SSMs offer a conceptually grounded and operationally transparent alternative to classical optimisation frameworks by translating these principles into computational terms, particularly in environments where the state space is too large to be evaluated analytically.

In addition to validating the feasibility of the approach, the model contributes to broader AI debates and highlights the value of biology-inspired assessment mechanisms as complements rather than substitutes for existing learning paradigms. While deep reinforcement, planning, and logic learning systems face difficulties in stochastic and high-dimensional domains, SSMs provide a fast, interpretable, and computationally cost-effective means of regulating behaviour. The emergence of rudimentary synthetic intuition suggests that biologically inspired evaluative heuristics, which have long been dismissed as irrational or secondary, may play an important role as a basic layer of adaptive intelligence, at least in simple artificial systems. However, their broader relevance for natural and more complex artificial agents remains an open question [6].

In summary, this study provides a starting point for investigating minimalist and bio-inspired evaluative architectures capable of improving artificial systems’ efficiency, transparency, and adaptability in scenarios of high uncertainty [10].

## 7. Directions for Future Research

The presented model is a conceptual prototype whose evolution offers multiple promising research opportunities. Future studies will progressively scale the environment (e.g., larger grids and structured tasks) to test whether the qualitative patterns observed in this study survive in less constrained domains. From the outset, the scalability and generalisation of Pixelverse to larger grids, continuous state spaces or spaces with greater granularity will allow the evaluation of the robustness of SSMs in more complex and realistically challenging contexts [25].

Beyond the minimal Pixelverse case study, a natural next step is to embed SSMs into standard grid-world benchmarks used in RL, comparing agents with and without the evaluative layer on families of stochastic navigation tasks [32,33]. Then, the performance could be assessed in terms of sample efficiency, environmental perturbation robustness, and learned policy stability, providing a systematic empirical contrast between somatic-marker-inspired evaluative mechanisms and conventional reward-driven baselines.

Another line of research may involve the integration of SSMs with RL methods, exploring synthetic valence as a reward signal, exploratory bias, or uncertainty modulator [6,8]. This hybridisation can bring artificial agents closer to contemporary neuromorphic and cognitive models by combining functional evaluative heuristics with advanced planning and optimisation strategies.

The development of multidimensional synthetic affects also represents a possibility of significant advancement, incorporating dimensions such as excitement, novelty, salience, and motivation, expanding behavioural expressiveness. We also acknowledge that the present one-dimensional valence signal is a deliberately simplified abstraction and that future SSM extensions should incorporate multidimensional synthetic affects (e.g., valence–arousal and additional evaluative axes such as novelty, salience, or motivation) so that richer trade-offs in complex decision-making contexts can be represented and analysed. This extension of research will enable the study of emerging artificial emotional dynamics, while maintaining the distinction between computational signals and biological experiences [10].

Further extensions will involve combining SSMs with symbolic modules, causal reasoning, and explicit memory, investigating the hypothesis that artificial agents can benefit from functional complementarity between implicit evaluative heuristics and explicit deliberative processes.

In addition, the computational lightness of SSMs makes them ideal candidates for applications in robotics and embedded systems, where rapid regulation and response to physical uncertainty skills are essential [12,19,34]. Recent bio-inspired hardware developments, including FPGA-based implementations of spiking neural networks for character recognition, illustrate how neuromorphic architectures can support efficient, low-power computation that is compatible with lightweight evaluative mechanisms, such as SSMs [35,36].

The potential compatibility with neuromorphic implementations also paves the way for the development of bio-inspired hardware dedicated to evaluative functions [21].

These lines of research contribute to the emergence of hybrid paradigms, where bioinspired evaluative heuristics contribute to more efficient and robust systems [37]. Advances in this line of research show that minimalist evaluation mechanisms have a strong potential to constitute building blocks in biologically inspired AI [38,39,40].

## Figures and Tables

**Table 1:** Main Differences Between Affective Models.

Dimension	Affective Computing [9]	Emotions in RL [12]	SSMs
Focus	Recognition and modelling of human emotions in hemiplegic patients.	Integrating emotion into the learning and action dynamics of RL agents.	Minimum heuristic evaluation for decision-making under combinatorial explosion.
Ticket Type	Multimodal: facial expression, voice, physiology, and context.	The agent’s states, rewards, values, and homeostatic variables.	Compressed signatures of the Pixelverse states.
Affective representation	Distinct categories or valence–activation spaces.	Emotional variables derived from the reward/value (e.g., fear, hope).	Single-valence scalar in the interval [−1, 1].
Role in the system	Improve human–machine interaction and user adaptation.	Modulation of learning, exploration and social behaviour of the agent.	Restrict the decision space (keep vs. reset) through accumulated evaluative markers.
Focus	Recognition and modelling of human emotions in hemiplegic patients.	Integrating emotion into the learning and action dynamics of RL agents.	Minimum heuristic evaluation for decision-making under combinatorial explosion.
Ticket Type	Multimodal: facial expression, voice, physiology, and context.	The agent’s states, rewards, values, and homeostatic variables.	Compressed signatures of the Pixelverse states.

**Table 2:** Summary of repeated-run metrics (30 runs, 100 iterations each).

Means	Mean ± SD
Total resets per run	1.7 ± 2.7
Proportion of “good” states (active ≤ 3)	0.26 ± 0.07
Average valence (3 most visited signatures)	−0.38 ± 0.22

**Table 3:** Performance of SSM and baseline agents in the 3 × 3 Pixelverse (30 runs, each with 100 iterations).

Agent Type	Total Resets per Run (Mean ± SD)	Proportion of “Good” States (Mean ± SD)	Notes
SSM agent	1.7 ± 1.96	0.24 ± 0.06	Single scalar valence
Random policy agent	20.93 ± 2.93	0.24± 0.05	Calibrated reset probability
Frequency-based agent (no SSM)	100.00 ± 0.00	0.00 ± 0.00	Empirical good-frequency rule
Q-learning agent	43.23 ± 4.03	0.15 ± 0.05	Tabular Q-learning (keep/reset)

## Data Availability

All data supporting the findings are publicly accessible via repository link to be added upon acceptance.

## Supplementary Materials

The following supporting information can be downloaded at: https://www.mdpi.com/article/10.3390/biomimetics11010063/s1, S1: Python computational simulation; S2: Baseline agents and comparison scripts.
