# Supplementary material for: Biomimetic Synthetic Somatic Markers in the Pixelverse: A Bio-Inspired Framework for Intuitive Artificial Intelligence
Source: Biomimetics (Basel). 2026 Jan 12;11(1):63. doi: 10.3390/biomimetics11010063 (PMC12838925; doi:10.3390/biomimetics11010063)
Supplement: Supplementary file 1 [file biomimetics-11-00063-s001.zip › biomimetics-4055609-supplementary.pdf]

Supplementary Material

S1: Python Computational simulation

```
# Authors: Vitor Lima & Domingos Martinho (2025)
# Description:
# Minimal reproducible simulation used in Section 3.
# Implements:
#    - Pixelverse environment
#   - Synthetic Somatic Marker (SSM)
#    - Agent with valence-based decision rule
# -------------------------------------------------------
import random
from dataclasses import dataclass, field
# -------------------------------------------------------
# Pixelverse Environment
# -------------------------------------------------------
@dataclass
class Pixelverse:
    size: int = 3
    flip_prob: float = 0.30
    def reset(self):
        """Generate a random grid of 0/1 values."""
        return [[random.randint(0, 1) for _ in range(self.size)]
                for _ in range(self.size)]
    def step(self, grid):
        """Evolve the grid by flipping cells with probability p."""
        new_grid = []
        for row in grid:
            new_row = []
            for cell in row:
                if random.random() < self.flip_prob:
                    new_row.append(1 - cell)
                else:
                    new_row.append(cell)
            new_grid.append(new_row)
        return new_grid
    @staticmethod
    def compress(grid):
        """State compression: count active pixels in each row."""
        return tuple(sum(row) for row in grid)
# -------------------------------------------------------
# Synthetic Somatic Marker (SSM)
# -------------------------------------------------------
@dataclass
class SyntheticSomaticMarker:
    alpha: float = 0.1
    memory: dict = field(default_factory=dict)
```

```
    def get_valence(self, signature):
        """Retrieve valence for a compressed state."""
        return self.memory.get(signature, 0.0)
    def update(self, signature, outcome):
        """
        outcome: +1 for good, -1 for bad
        """
        v = self.get_valence(signature)
        v += self.alpha * outcome
        v = max(min(v, 1.0), -1.0)
        self.memory[signature] = v
# --------------------------------------------------------
# Agent
# --------------------------------------------------------
@dataclass
class Agent:
    threshold: float = -0.5  # below this → reset
    def decide(self, valence):
        """Return action: 'keep' or 'reset'."""
        return "reset" if valence < self.threshold else "keep"
# --------------------------------------------------------
# Simulation
# --------------------------------------------------------
def run_simulation(iterations=20, verbose=True):
    px = Pixelverse()
    ssm = SyntheticSomaticMarker()
    agent = Agent()
    grid = px.reset()
    log = []
    for t in range(iterations):
        signature = px.compress(grid)
        valence = ssm.get_valence(signature)
        action = agent.decide(valence)
        if action == "reset":
            grid = px.reset()
            outcome = -1
        else:
            grid = px.step(grid)
            # Example outcome rule: grids with <= 3 active cells are "good"
            active = sum(sum(row) for row in grid)
            outcome = +1 if active <= 3 else -1
        ssm.update(signature, outcome)
        log.append((t, signature, valence, action, outcome))
        if verbose:
            print(f"t={t}, sig={signature}, V={valence:.2f}, "
                  f"act={action}, R={outcome}")
    return log, ssm.memory
```

S2: Baseline agents and comparison

```
# Authors: Vitor Lima & Domingos Martinho (2025)
# Description:
# Minimal reproducible simulation used in Section 4.2.
# ----------------------------------------------------------
import random
from dataclasses import dataclass, field
import math
@dataclass
class Pixelverse:
    size: int = 3
    flip_prob: float = 0.30
    def reset(self):
        return [[random.randint(0, 1) for _ in range(self.size)]
                for _ in range(self.size)]
    def step(self, grid):
        new_grid = []
        for row in grid:
            new_row = []
            for cell in row:
                if random.random() < self.flip_prob:
                    new_row.append(1 - cell)
                else:
                    new_row.append(cell)
            new_grid.append(new_row)
        return new_grid
    @staticmethod
    def compress(grid):
        return tuple(sum(row) for row in grid)
# ----------------------------------------------------------
# Synthetic Somatic Marker
# ----------------------------------------------------------
@dataclass
class SyntheticSomaticMarker:
    alpha: float = 0.1
    memory: dict = field(default_factory=dict)
    def get_valence(self, signature):
        return self.memory.get(signature, 0.0)
    def update(self, signature, outcome):
        v = self.get_valence(signature)
        v += self.alpha * outcome
        v = max(min(v, 1.0), -1.0)
        self.memory[signature] = v
# ----------------------------------------------------------
# Agent for SSM
# ----------------------------------------------------------
```

```
@dataclass
class AgentSSM:
    threshold: float = -0.5  # below this → reset
    def decide(self, valence):
        return "reset" if valence < self.threshold else "keep"
# --------------------------------------------------------
# Random policy agent
# --------------------------------------------------------
@dataclass
class AgentRandom:
    p_reset: float = 0.2  # can be adjusted if needed
    def decide(self, _signature=None, _valence=None):
        return "reset" if random.random() < self.p_reset else "keep"
# --------------------------------------------------------
# Frequency-based agent (no valence accumulation)
# --------------------------------------------------------
@dataclass
class AgentFrequency:
    threshold: float = 0.5
    counts: dict = field(default_factory=dict)  # signature -> (good, bad)
    def get_freq_good(self, signature):
        good, bad = self.counts.get(signature, (0, 0))
        total = good + bad
        return good / total if total > 0 else 0.0
    def update(self, signature, outcome):
        # outcome: +1 good, -1 bad
        good, bad = self.counts.get(signature, (0, 0))
        if outcome == 1:
            good += 1
        else:
            bad += 1
        self.counts[signature] = (good, bad)
    def decide(self, signature):
        freq_good = self.get_freq_good(signature)
        return "keep" if freq_good > self.threshold else "reset"
# --------------------------------------------------------
# Tabular Q-learning agent (keep / reset)
# --------------------------------------------------------
@dataclass
class AgentQLearning:
    alpha: float = 0.1
    gamma: float = 0.95
    epsilon: float = 0.1
    q: dict = field(default_factory=dict)  # (signature, action) -> value
    def get_q(self, signature, action):
        return self.q.get((signature, action), 0.0)
    def choose_action(self, signature):
```

```
        if random.random() < self.epsilon:
            return random.choice(["keep", "reset"])
        q_keep = self.get_q(signature, "keep")
        q_reset = self.get_q(signature, "reset")
        if q_keep > q_reset:
            return "keep"
        elif q_reset > q_keep:
            return "reset"
        else:
            return random.choice(["keep", "reset"])
    def update(self, signature, action, reward, next_signature):
        q_sa = self.get_q(signature, action)
        q_keep_next = self.get_q(next_signature, "keep")
        q_reset_next = self.get_q(next_signature, "reset")
        max_next = max(q_keep_next, q_reset_next)
        target = reward + self.gamma * max_next
        new_q = q_sa + self.alpha * (target - q_sa)
        self.q[(signature, action)] = new_q
# --------------------------------------------------------
# Helper: run one episode for a generic "agent type"
# --------------------------------------------------------
def run_episode_ssm(iterations=100):
    px = Pixelverse()
    ssm = SyntheticSomaticMarker()
    agent = AgentSSM()
    grid = px.reset()
    resets = 0
    good_states = 0
    for t in range(iterations):
        signature = px.compress(grid)
        valence = ssm.get_valence(signature)
        action = agent.decide(valence)
        if action == "reset":
            grid = px.reset()
            outcome = -1
            resets += 1
        else:
            grid = px.step(grid)
            active = sum(sum(row) for row in grid)
            outcome = 1 if active <= 3 else -1
        if outcome == 1:
            good_states += 1
        ssm.update(signature, outcome)
    return resets, good_states / iterations
def run_episode_random(iterations=100, p_reset=0.2):
    px = Pixelverse()
    agent = AgentRandom(p_reset=p_reset)
```

```
    grid = px.reset()
    resets = 0
    good_states = 0
    for t in range(iterations):
        signature = px.compress(grid)
        action = agent.decide()
        if action == "reset":
            grid = px.reset()
            reward = -1
            resets += 1
        else:
            grid = px.step(grid)
            active = sum(sum(row) for row in grid)
            reward = 1 if active <= 3 else -1
        if reward == 1:
            good_states += 1
    return resets, good_states / iterations
def run_episode_frequency(iterations=100, threshold=0.5):
    px = Pixelverse()
    agent = AgentFrequency(threshold=threshold)
    grid = px.reset()
    resets = 0
    good_states = 0
    for t in range(iterations):
        signature = px.compress(grid)
        action = agent.decide(signature)
        if action == "reset":
            grid = px.reset()
            outcome = -1
            resets += 1
        else:
            grid = px.step(grid)
            active = sum(sum(row) for row in grid)
            outcome = 1 if active <= 3 else -1
        if outcome == 1:
            good_states += 1
        agent.update(signature, outcome)
    return resets, good_states / iterations
def run_episode_qlearning(iterations=100, alpha=0.1, gamma=0.95, epsilon=0.1):
    px = Pixelverse()
    agent = AgentQLearning(alpha=alpha, gamma=gamma, epsilon=epsilon)
    grid = px.reset()
    resets = 0
    good_states = 0
    signature = px.compress(grid)
    for t in range(iterations):
        action = agent.choose_action(signature)
```

```
        if action == "reset":
            grid = px.reset()
            reward = -1
            resets += 1
        else:
            grid = px.step(grid)
            active = sum(sum(row) for row in grid)
            reward = 1 if active <= 3 else -1
        if reward == 1:
            good_states += 1
        next_signature = px.compress(grid)
        agent.update(signature, action, reward, next_signature)
        signature = next_signature
    return resets, good_states / iterations
# --------------------------------------------------------
# Utility to compute mean ± SD
# --------------------------------------------------------
def mean_sd(values):
    n = len(values)
    m = sum(values) / n
    var = sum((v - m) ** 2 for v in values) / n
    return m, math.sqrt(var)
# --------------------------------------------------------
# Main experiment: 30 runs × 100 iterations per agent
# --------------------------------------------------------
def main():
    runs = 30
    iters = 100
    # 1) SSM agent
    resets_ssm = []
    good_ssm = []
    for _ in range(runs):
        r, g = run_episode_ssm(iterations=iters)
        resets_ssm.append(r)
        good_ssm.append(g)
    m_r_ssm, sd_r_ssm = mean_sd(resets_ssm)
    m_g_ssm, sd_g_ssm = mean_sd(good_ssm)
    # 2) Random agent (ajusta p_reset se quiseres)
    resets_rand = []
    good_rand = []
    for _ in range(runs):
        r, g = run_episode_random(iterations=iters, p_reset=0.2)
        resets_rand.append(r)
        good_rand.append(g)
    m_r_rand, sd_r_rand = mean_sd(resets_rand)
    m_g_rand, sd_g_rand = mean_sd(good_rand)
    # 3) Frequency-based agent
```

```
    resets_freq = []
    good_freq = []
    for _ in range(runs):
        r, g = run_episode_frequency(iterations=iters, threshold=0.5)
        resets_freq.append(r)
        good_freq.append(g)
    m_r_freq, sd_r_freq = mean_sd(resets_freq)
    m_g_freq, sd_g_freq = mean_sd(good_freq)
    # 4) Q-learning agent
    resets_q = []
    good_q = []
    for _ in range(runs):
        r, g = run_episode_qlearning(iterations=iters, alpha=0.1, gamma=0.95, epsilon=0.1)
        resets_q.append(r)
        good_q.append(g)
    m_r_q, sd_r_q = mean_sd(resets_q)
    m_g_q, sd_g_q = mean_sd(good_q)
    print("Agent\tResets (mean±SD)\tGood proportion (mean±SD)")
    print(f"SSM\t{m_r_ssm:.2f} ± {sd_r_ssm:.2f}\t\t{m_g_ssm:.2f} ± {sd_g_ssm:.2f}")
    print(f"Random\t{m_r_rand:.2f} ± {sd_r_rand:.2f}\t\t{m_g_rand:.2f} ±
{sd_g_rand:.2f}")
    print(f"Freq\t{m_r_freq:.2f} ± {sd_r_freq:.2f}\t\t{m_g_freq:.2f} ± {sd_g_freq:.2f}")
    print(f"Q-learn\t{m_r_q:.2f} ± {sd_r_q:.2f}\t\t{m_g_q:.2f} ± {sd_g_q:.2f}")

if __name__ == "__main__":
    main()
```
